# *TIMP-2* SNPs rs7342880 and rs4789936 are linked to risk of knee osteoarthritis in the Chinese Han Population

**DOI:** 10.18632/oncotarget.13590

**Published:** 2016-11-25

**Authors:** Pengcheng Xu, Wen Guo, Tianbo Jin, Jihong Wang, Dongsheng Fan, Zengtao Hao, Shangfei Jing, ChaoQian Han, Jieli Du, Dong Jiang, Shuzheng Wen, Jianzhong Wang

**Affiliations:** ^1^ Inner Mongolia Medical University, Hohhot 010010, Inner Mongolia, China; ^2^ Key Laboratory of Resource Biology and Biotechnology in Western China (Northwest University), Ministry of Education, School of Life Sciences, Northwest University, Xi'an, Shaanxi 710069, China; ^3^ Xi'an Tiangen Precision Medical Institute, Xi'an, Shaanxi, 710075, China; ^4^ Department of Hand and Foot Surgery, Second Affiliated Hospital, Inner Mongolia Medical University, Hohhot 010030, Inner Mongolia Autonomous Region, China; ^5^ Department of Trauma, Second Affiliated Hospital, Inner Mongolia Medical University, Hohhot 010030, Inner Mongolia Autonomous Region, China

**Keywords:** knee osteoarthritis, *TIMP-2*, single nucleotide polymorphism, gene

## Abstract

This study aimed to investigate whether functional polymorphisms in the *tissue inhibitors of metalloproteinase-2* (*TIMP-2*) gene are associated with susceptibility to knee osteoarthritis (OA) in the Chinese Han population. Six *TIMP-2* single nucleotide polymorphisms (SNPs) were assayed using MassARRAY in 300 patients clinically and radiographically diagnosed with knee OA and in 428 controls. Allelic and genotypic frequencies were compared between groups. Logistic regression adjusting for age and gender was used to estimate risk associations between specific genotypes and knee OA by computing odds ratios (ORs) and 95% confidence intervals (95% CIs). We found that allele “A” in rs7342880 was significantly associated with increased risk of knee OA (OR = 1.44, 95%CI = 1.09-1.91, *p* = 0.035). In addition, in the over-dominant model, rs4789936 correlated with reduced risk of knee OA, adjusting for age and gender (OR = 0.69, 95%CI = 0.49-0.98, *p* = 0.036). Finally, rs7342880 correlated with increased risk of knee OA in females. This study provides evidence that *TIMP-2* is a knee OA susceptibility gene in the Chinese population and a potential diagnostic and preventive marker for the disease.

## INTRODUCTION

Knee osteoarthritis (OA) is the most common type of arthritis and a leading cause of chronic disability. It affects 10% of men and 18% of women over 60 years of age and leads to substantial cost to the individual and society [1–3]. The disease is characterized by morphological, biochemical, molecular, and biomechanical changes of both cells and extracellular matrix (ECM) which lead to a slowly progressive loss of articular cartilage, sclerosis of subchondral bone, osteophytes, and subchondral cysts [4]. Metalloproteinase family proteins (MMPs) control ECM integrity by catalyzing the degradation of structural proteins including laminin, fibronectin, and various types of collagen [5, 6]. The degradation of structural proteins in cartilage, which is a feature of arthritic diseases, is accelerated by increased expression of MMPs by chondrocytes [7]. On the other hand, tissue inhibitors of metalloproteases (TIMPs) inhibit the activity of metalloproteinases by interacting with the MMP active site [8]. Therefore, osteoarthritic cartilage is characterized by an imbalance between MMPs and TIMPs.

Although the etiology and pathogenesis of knee OA remain unclear, some factors that may increase the risk of knee OA are undisputed, such as gender, advanced age, ethnicity, behavioral influences, obesity, occupation, and previous injury [9–11]. Furthermore, genetic variation remains the strongest predictor of the onset and progression of this disease [12–15].

Bhumsuk Keam et al. [16] identified the SNP rs4789934 in the *TIMP-2* gene as a potential susceptibility locus for knee OA (*p* = 4.01 × 10^−6^) in a GWAS of 3,793 samples (476 cases: wrist + knee and 3317 controls). Jing-Bo Ji et al. [17] confirmed that the expression of serum *TIMP-2* is significantly decreased in an animal model of OA. Fang-Jie Zhang et al. [18] carried out an *in vitro* experimental study using tissue samples from 16 Chinese patients with knee OA and identified mRNA expression of *TIMP-1* and *TIMP-2* significantly increased cytotoxicity or apoptosis of chondrocytes. Additionally, Yiqian Liang et al. [19] found no significant association for rs2277698 in *TIMP-2* gene with knee OA in another case-control study involving a Korean population. To date, no studies have assessed the correlation between SNPs in *TIMP-2* and the risk of knee OA in the Chinese Han population. In the present case-control study, we evaluated the effect of *TIMP-2* polymorphisms on knee OA in the Chinese Han population, based on the findings of the GWAS using populations of Korean ancestry.

## RESULTS

### Participant characteristics

The demographic characteristics of the study population, including gender, age, and course of disease, are summarized in Table 1. The study included 300 OA cases (100 male and 200 female) and 428 controls (197 male and 231 female). The mean age of the patients and the control group were 52.71 ± 8.762 years (range: 47-72 years) and 60.64 ± 4.822 years (range: 41-75 years), respectively. OA patients were divided into two groups based on the time course of their disease (< 10 years & ≥10 years), and there were significant differences between these groups in age (*p* < 0.001) and gender (*p* = 0.001).

**Table 1:** Characteristics of the study subjects

Characteristics	Cases (n = 300)	Controls (n = 428)	*p*^a^
Gender			
Female	200	231	0.001^*^
Male	100	197	
Age (mean ± SD years)	52.71±8.762	60.64±4.822	< 0.001^*^
Course of disease (years)			
< 10	163		
≥ 10	137		

* *p* < 0.05 indicates statistical significance

a Two-sided Chi-squared test

### Association between *TIMP-2* polymorphisms and knee OA risk

The basic information related to candidate SNPs in our study such as chromosomal position, gene, allele, HWE test results, and minor allele frequency (MAF) appear in Table 2. We assumed that the minor allele of each SNP was a risk allele compared to the wild-type allele. We used χ^2^ test to compare the differences in frequency distributions of alleles between cases and controls and found just one significant SNP in the *TIMP-2* gene at a 5% level (allele “A” in rs7342880, *p* = 0.011, OR = 1.44, 95%CI = 1.09-1.91). Rs2003241 was excluded for significant deviation from Hardy-Weinberg Equilibrium (HWE) (*p* < 0.05). The HWE in other SNPs in the control group was similar to those of the HapMap Asian population (http://hapmap.ncbi.nlm.nih.gov/).

**Table 2:** Candidate SNPs examined in ***TIMP-2*** gene

SNP	Gene Name	Chromosome Position	Base Change	MAF-Case	MAF-Control	*p* value for HWE test	OR	95%CI		p^*^
rs2277698	*TIMP2*	17q25.3	T/C	0.21	0.23	0.58	0.88	0.68	1.13	0.314
rs2009196	*TIMP2*	17q25.3	C/G	0.44	0.41	0.49	1.11	0.90	1.37	0.320
rs7342880	*TIMP2*	17q25.3	A/C	0.19	0.14	0.55	1.44	1.09	1.91	0.011^*^
rs11654470	*TIMP2*	17q25.3	C/T	0.25	0.27	0.54	0.88	0.70	1.12	0.308
rs2003241	*TIMP2*	17q25.3	C/T	0.19	0.16	0.03^*^	1.22	0.93	1.59	0.159
rs4789936	*TIMP2*	17q25.3	T/C	0.28	0.30	1.00	0.89	0.71	1.12	0.327

SNP = single nucleotide polymorphism; MAF = minor allele frequency; OR = odds ratio; 95 % CI = 95 % confidence interval;

HWE = Hardy–Weinberg equilibrium;

* *p* < 0.05 indicates statistical significance;

*p* value were calculated using two-sided Chi-squared test.

The association results between *TIMP-2* SNPs and risk of knee OA under the genetic model are listed in Table 3. Before adjustment, we identified the minor “A” allele in rs7342880 was associated with an increased risk of knee OA based on analysis using the co-dominant, dominant, and additive models (*p* < 0.05 for all) in Table 4. After adjustment for gender and age, the over-dominant model showed that the rs4789936 SNP was significantly associated with a 0.69-fold decreased OA risk (*p* < 0.05). Nevertheless, the genetic model in rs7342880 showed no significant difference between OA patients and controls (*p* > 0.05) after adjustment. In our statistical analysis, we found no statistically significant associations between SNPs and risk of knee OA after Bonferroni correction. This may be due to the relatively small sample size or the weakness of Bonferroni correction itself (the interpretation of a finding depends on the number of other tests performed).

**Table 3:** Frequency distributions of SNPs in ***TIMP-2*** and their associations with the risk of developing knee OA under multiple models of inheritance

SNP	Model	Genotype	Control N (%)	Case N (%)	Adjustment analysis		Crude analysis	
					OR (95% CI)	*p*^a^	OR (95% CI)	*p* ^b^
rs2277698	Codominant	C/C	251 (58.9%)	187 (62.3%)	1	0.400	1	0.590
		C/T	155 (36.4%)	102 (34%)	0.82 (0.57-1.17)		0.88 (0.65-1.21)	
		T/T	20 (4.7%)	11 (3.7%)	0.66 (0.28-1.59)		0.74 (0.35-1.58)	
	Dominant	C/C	251 (58.9%)	187 (62.3%)	1	0.210	1	0.350
		C/T-T/T	175 (41.1%)	113 (37.7%)	0.80 (0.56-1.13)		0.87 (0.64-1.17)	
	Recessive	C/C-C/T	406 (95.3%)	289 (96.3%)	1	0.450	1	0.500
		T/T	20 (4.7%)	11 (3.7%)	0.72 (0.30-1.70)		0.77 (0.36-1.64)	
	Over-dominant	C/C-T/T	271 (63.6%)	198 (66%)	1	0.330	1	0.510
		C/T	155 (36.4%)	102 (34%)	0.84 (0.59-1.19)		0.90 (0.66-1.23)	
	Log-additive	---	---	---	0.82 (0.61-1.10)	0.180	0.87 (0.67-1.13)	0.310
rs2009196	Codominant	G/G	151 (35.4%)	94 (31.3%)	1	0.880	1	0.530
		C/G	200 (46.8%)	149 (49.7%)	1.07 (0.73-1.57)		1.20 (0.86-1.67)	
		C/C	76 (17.8%)	57 (19%)	0.96 (0.58-1.59)		1.20 (0.78-1.85)	
	Dominant	G/G	151 (35.4%)	94 (31.3%)	1	0.830	1	0.260
		C/G-C/C	276 (64.6%)	206 (68.7%)	1.04 (0.72-1.50)		1.20 (0.88-1.64)	
	Recessive	G/G-C/G	351 (82.2%)	243 (81%)	1	0.720	1	0.680
		C/C	76 (17.8%)	57 (19%)	0.92 (0.59-1.43)		1.08 (0.74-1.58)	
	Over-dominant	G/G-C/C	227 (53.2%)	151 (50.3%)	1	0.640	1	0.450
		C/G	200 (46.8%)	149 (49.7%)	1.08 (0.77-1.52)		1.12 (0.83-1.51)	
	Log-additive	---	---	---	0.99 (0.78-1.27)	0.950	1.11 (0.90-1.37)	0.320
rs7342880	Codominant	C/C	318 (74.3%)	199 (66.3%)	1	0.180	1	0.046^*^
		C/A	100 (23.4%)	88 (29.3%)	1.29 (0.88-1.90)		1.41 (1.00-1.97)	
		A/A	10 (2.3%)	13 (4.3%)	2.02 (0.77-5.32)		2.08 (0.89-4.83)	
	Dominant	C/C	318 (74.3%)	199 (66.3%)	1	0.110	1	0.020^*^
		C/A-A/A	110 (25.7%)	101 (33.7%)	1.36 (0.94-1.96)		1.47 (1.06-2.03)	
	Recessive	C/C-C/A	418 (97.7%)	287 (95.7%)	1	0.190	1	0.130
		A/A	10 (2.3%)	13 (4.3%)	1.88 (0.72-4.92)		1.89 (0.82-4.38)	
	Over-dominant	C/C-A/A	328 (76.6%)	212 (70.7%)	1	0.250	1	0.071
		C/A	100 (23.4%)	88 (29.3%)	1.25 (0.85-1.83)		1.36 (0.97-1.90)	
	Log-additive	---	---	---	1.34 (0.98-1.84)	0.069	1.42 (1.08-1.87)	0.013^*^
rs11654470	Codominant	T/T	224 (52.3%)	168 (56%)	1	0.250	1	0.580
		T/C	175 (40.9%)	115 (38.3%)	0.78 (0.55-1.12)		0.88 (0.64-1.19)	
		C/C	29 (6.8%)	17 (5.7%)	0.63 (0.31-1.31)		0.78 (0.42-1.47)	
	Dominant	T/T	224 (52.3%)	168 (56%)	1	0.120	1	0.330
		T/C-C/C	204 (47.7%)	132 (44%)	0.76 (0.54-1.07)		0.86 (0.64-1.16)	
	Recessive	T/T-T/C	399 (93.2%)	283 (94.3%)	1	0.330	1	0.540
		C/C	29 (6.8%)	17 (5.7%)	0.71 (0.35-1.43)		0.83 (0.45-1.53)	
	Over-dominant	T/T-C/C	253 (59.1%)	185 (61.7%)	1	0.260	1	0.490
		T/C	175 (40.9%)	115 (38.3%)	0.82 (0.58-1.16)		0.90 (0.66-1.22)	
	Log-additive	---	---	---	0.79 (0.60-1.04)	0.096	0.88 (0.69-1.12)	0.300
rs2003241	Codominant	T/T	305 (71.3%)	197 (65.7%)	1	0.520	1	0.580
		T/C	105 (24.5%)	90 (30%)	1.24 (0.84-1.81)		0.88 (0.64-1.19)	
		C/C	18 (4.2%)	13 (4.3%)	0.91 (0.40-2.08)		0.78 (0.42-1.47)	
	Dominant	T/T	305 (71.3%)	197 (65.7%)	1	0.360	1	0.110
		T/C-C/C	123 (28.7%)	103 (34.3%)	1.18 (0.82-1.70)		1.30 (0.94-1.78)	
	Recessive	T/T-T/C	410 (95.8%)	287 (95.7%)	1	0.710	1	0.930
		C/C	18 (4.2%)	13 (4.3%)	0.86 (0.38-1.94)		1.03 (0.50-2.14)	
	Over-dominant	T/T-C/C	323 (75.5%)	210 (70%)	1	0.260	1	0.100
		T/C	105 (24.5%)	90 (30%)	1.24 (0.85-1.82)		1.32 (0.95-1.84)	
	Log-additive	---	---	---	1.10 (0.81-1.48)	0.540	1.20 (0.92-1.56)	0.180
rs4789936	Codominant	C/C	208 (48.6%)	160 (54%)	1	0.110	1	0.250
		C/T	181 (42.3%)	107 (36.1%)	0.68 (0.47-0.98)		0.77 (0.56-1.05)	
		T/T	39 (9.1%)	29 (9.8%)	0.92 (0.50-1.69)		0.97 (0.57-1.63)	
	Dominant	C/C	208 (48.6%)	160 (54%)	1	0.058	1	0.150
		C/T-T/T	220 (51.4%)	136 (46%)	0.72 (0.51-1.01)		0.80 (0.60-1.08)	
	Recessive	C/C-C/T	389 (90.9%)	267 (90.2%)	1	0.790	1	0.760
		T/T	39 (9.1%)	29 (9.8%)	1.09 (0.60-1.96)		1.08 (0.65-1.80)	
	Over-dominant	C/C-T/T	247 (57.7%)	189 (63.9%)	1	0.036^*^	1	0.096
		C/T	181 (42.3%)	107 (36.1%)	0.69 (0.49-0.98)		0.77 (0.57-1.05)	
	Log-additive	---	---	---	0.84 (0.64-1.09)	0.180	0.89 (0.71-1.12)	0.340

* *p* < 0.05 indicates statistical significance; OR = odds ratio; 95 % CI = 95 % confidence interval;

a *p* values were calculated by unconditional logistic regression adjusted for age and gender

b *p* values were calculated without adjustment for age and gender

**Table 4:** Frequency distributions of rs7342880 SNP and its association with the risk of developing knee OA

Variable	rs7342880				
	CC	CA+AA	*p*	OR	95%/CI
Age (year)					
> 60	101	51	---	1.00	(reference)
≤ 60	98	50	0.966	0.99	0.613-1.598
Course of disease (year)					
< 10	112	51	---	1.00	(reference)
≥10	87	50	0.342	1.262	0.781-2.040
Gender					
Male	74	26	---	1.00	(reference)
Female	125	75	0.048^*^	1.708	1.004-2.903

OR = odds ratio; 95 % CI = 95 % confidence interval;

* *p* < 0.05 indicates statistical significance;

*p* value were calculated using two-sided Chi-squared test

### Association between *TIMP-2* polymorphisms and knee OA patient clinicopathological features

We investigated the interactions between clinical characteristics and the SNPs. Gender was the only significant variable, and among females the genotype “CA+AA” in rs7342880 SNP was associated with 1.708-fold increased odds of knee OA, compared to genotype CC, adjusting for age and gender (adjusted OR = 1.708, *p* = 0.048, 95% CI= 1.004-2.903) (Table 4).

### Haplotype analysis

**Figure 1:**
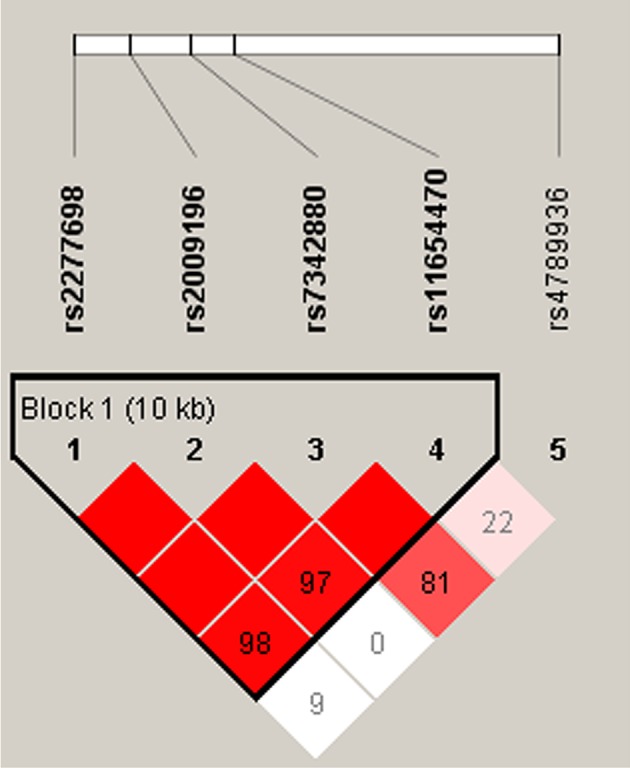
linkage disequilibrium of polymorphic sites in the *TIMP-2* gene

One block (rs2277698 and rs11654470) was detected in the *TIMP-2* SNPs by haplotype analyses (Figure 1). The results of the association between the *TIMP-2* haplotype and the risk of knee OA are listed in Table 5. In this block, haplotype “CCAT” was associated with risk of knee OA (OR = 1.39; 95%CI = 1.04–1.86;*p* = 0.025). However, when adjusted by gender and age, there was no association between this *TIMP-2* haplotype and the risk of knee OA (*p* > 0.05).

**Table 5:** ***TIMP-2*** haplotype frequencies and the association with knee OA risk in case and control subjects

rs2277698	rs2009196	rs7342880	rs11654470	Frequence (%)		χ^2^	Crude Analysis		Adjusted Analysis	
				Case	Control		OR (95% CI)	*p*^a^	OR (95% CI)	*p*^b^
C	G	C	T	0.564	0.584	0.632	1	---	1	---
T	C	C	C	0.207	0.229	0.916	0.93 (0.71 - 1.22)	0.620	0.85 (0.62 - 1.16)	0.310
C	C	A	T	0.187	0.14	5.949	1.39 (1.04 - 1.86)	0.025^*^	1.27 (0.91 - 1.77)	0.160
C	C	C	C	0.04	0.041	0.015	0.99 (0.57 - 1.71)	0.960	0.74 (0.39 - 1.39)	0.350

OR = odds ratio; 95 % CI = 95 % confidence interval;

* *p* < 0.05 indicates statistical significance;

a *p* value from were calculated from two-sided Chi-squared test without adjusted by age and gender;

b *p* values were calculated by unconditional logistic regression adjusted for age and sex.

### Stratified analysis

In stratified analysis by gender, we found rs7342880 SNP was not significantly associated to knee OA in males (*p* > 0.05) but was statistically significant in females (*p* < 0.05) (Table 6). We took common genotypes as the reference in male or female controls. The “CA+ AA” genotype of rs7342880 was associated with increased knee OA risk in females (*p* = 0.048, OR = 1.55, 95%CI = 1.00-2.39). We detected no significant interactions between these and other SNPs examined (data not shown).

**Table 6:** Significant associations between SNPs and knee OA after stratification by gender

Genotype	Female						Male					
	case		control		OR (95 % CI)	*p*^a^	case		control		OR (95 % CI)	*p*^a^
	N	%	N	%			N	%	N	%		
C/C	125	62.5	171	74	1 [Ref]		74	74	147	74.6	1 [Ref]	
C/A	67	33.5	54	23.4	1.52 (0.97-2.39)	0.140	21	21	46	23.4	0.96 (0.44-2.06)	0.580
A/A	8	4	6	2.6	1.80 (0.58-5.52)		5	5	4	2	3.02 (0.35-25.91)	
C/A-A/A	75	37.5	60	26	1.55 (1.00-2.39)	0.048^*^	26	26	50	25.4	1.07 (0.51-2.22)	0.860

OR = odds ratio; 95 % CI = 95 % confidence interval; [Ref] = reference category;

* *p* < 0.05 indicates statistical significance;

a *p* value from were calculated from two-sided Chi-squared test adjusted by age and gender

## DISCUSSION

Cartilage degradation in knee OA involves MMP activity as collagenases (MMP-1 and 13), gelatinases (MMP-2 and MMP-9) and stromelysin (MMP-3) [20]. Of the three major MMPs that degrade native collagen (MMP-1, MMP-8, and MMP-13), recent work has shown MMP-13 was the most important in knee OA due to its preferential degradation of type II collagen [21]. Furthermore, increased expression of MMP-13 is observed in knee OA [22]. Nevertheless, all active MMPs are inhibited by TIMPs. TIMP-2 is a non-glycosylated protein, which blocks the hydrolase activity of all MMPs [23], including MMP-13. In addition, *in vitro* experiments demonstrated an association between TIMP-2 and osteopontin, involving pathological changes of knee OA [18].

*TIMP-2* gene polymorphisms have been studied in several diseases such as hypertension [24], Achilles tendon pathologies [25], cancer [26], and varicose vein [27]. In the current study, we studied six *TIMP-2* gene polymorphisms in Chinese patients with knee OA and healthy controls. We found that rs7342880, an intronic SNP within the *TIMP-2* gene, was significantly associated with knee OA risk according to allele association analysis. A similar distribution of other polymorphisms was observed in both studied groups. “A” allele of rs7342880 was ascertained to be associated with increased risk for the development of knee OA. Similar to our results, rs7342880 has also been associated with increased risk of knee OA in a previous study involving a Spanish population [28]. This study is the first genotype/allele-based study that describes the association between SNPs within the *TIMP-2* locus and knee OA risk in a Chinese population.

We found another polymorphism in *TIMP-2* rs4789936 associated with decreased risk of knee OA by the over-dominant model. A previous study found that rs4789936 SNP in *TIMP-2* gene may be involved in breast cancer susceptibility and survival in a Shanghai-based population [29]. Our study is the first report on the association between the rs4789936 SNP in *TIMP-2* and knee OA risk in any Chinese population. Further studies to determine the mechanism of rs4789936 should be considered.

Haplotype analysis suggested that knee OA risk was substantially elevated among individuals with specific haplotypes. Before adjustment, “CCAT” was a risk haplotype (OR = 1.39). After adjustment for age and gender, we found no statistically significant association between these *TIMP-2* SNPs and knee OA risk. The mechanism responsible for this finding is unclear. This trend did not reach statistical significance, perhaps because of the small number of patients included.

By stratification analysis in gender-specific populations, we found that the “CA+AA” genotype of rs7342880 was associated with increased risk of knee OA in females compared to the “C/C” genotype. Similarly, epidemiological studies have shown that females have an increased risk of OA occurrence in the hand, knee, and in multiple joints [30], which may help explain the sex differences in the prevalence of OA. In addition, gene polymorphisms were analyzed to establish their associations with clinicopathological features, including gender, age, and duration of knee OA (years). rs7342880 was associated with increased risk of knee OA (without adjustment, “CA+AA” OR = 1.708; 95%CI = 1.004-2.903; *p* = 0.048), consistent with stratification analysis. The accelerated rise in knee OA incidence in women following menopause suggests a possible role for sex hormones, particularly estrogen deficiency. However, there is no consistent evidence linking circulating sex hormone levels [31, 32] with OA prevalence. Therefore, the results of the association between gene polymorphisms and the risk of knee OA in females were stable, because we excluded the effects of sex hormones.

Some inherent limitations of this case–control study must be noted. First, the sample size is relatively small relative to knee OA association studies published to date [33–35]. Additionally, body mass index (BMI) is a known confounding factor for association analysis and affects the onset or progress of knee OA [36, 37]. In this study, knee OA patients and controls were not adjusted for BMI. In future studies, to uncover the role of BMI between genetic variants and the risk of knee OA, BMI should be recorded in the same OA subjects or even the more samples. In addition, the results of our population comparison by the χ^2^ test indicated that there are some differences in gender and age. Multiple independent studies with large sample sizes and gathering detailed clinical information are needed to validate our findings.

In conclusion, our comprehensive analysis, for the first time, suggests that SNPs (rs7342880 and rs4789936) in *TIMP-2* genotype and allele are associated with knee OA risk in Chinese Han population. Identifying the underlying genetic factors could enhance our understanding of the pathogenesis of this complex disorder, which could eventually contribute to improved prevention and treatment of this common disease. Further studies are needed in large cohorts of knee OA patients including the polymorphisms of other *TIMPs* (i.e., *TIMP*-1, *TIMP*-4) genes.

## MATERIALS AND METHODS

### Subject recruitment and sample collection

We recruited 300 Han Chinese patients diagnosed with knee OA for this case-control study from February 2013 to July 2015. All patients were diagnosed by the Second Department of Trauma & Hand and Foot Surgery, Second Affiliated Hospital, Inner Mongolia Medical University, China. The diagnosis of knee OA was based on a detailed history, physical exam, and/or radiographic studies. Diagnostic criteria developed by the American College of Rheumatology (ACR) include the presence of knee joint pain, osteophytes or bone spurs on X-ray, and one or more associated symptoms in the knee joint. Other associated symptoms include knee stiffness, crepitus on physical exam, point tenderness, enlargement or deformity of the knee joint, and a narrowing knee joint space noted on radiography [38]. In our study, we used broad inclusion criteria for primary knee OA cases with any symptoms and signs of OA, and radiographic signs of knee OA according to the Kellgren–Lawrence grading. Additionally, participants who had undergone total knee replacement with a radiological score > 2 on the Kellgren and Lawrence scale were included. Exclusion criteria were other etiologies causing knee diseases such as inflammatory arthritis (rheumatoid, polyarthritis, or autoimmune disease), post-traumatic or post-septic arthritis, skeletal dysplasia, or developmental dysplasia.

The control group included 428 individuals who had never exhibited any signs or symptoms of knee OA, other arthritis, or joint diseases (pain, swelling, tenderness, or restriction of movement) at any site, based on their medical history and a thorough examination conducted by an experienced physiatrist. In addition, patients who had undergone treatment for injuries and fractures, with no symptoms or clinical signs of OA were also included.

All individuals were of Chinese origin. This study was approved by the ethics committee of Inner Mongolia Medical University and written informed consent was obtained from all participants.

### SNP selection and genotyping

Genetic variants were randomly selected from the *TIMP-2* gene annotated by the HapMap database. Six candidate SNPs (rs2277698, rs2009196, rs7342880, rs11654470, rs2003241, rs4789936) in the *TIMP-2* gene with minor allele frequencies (MAF) >5 % in Asians were finally selected for genotyping. Five milliliters of venous blood were collected in ethylenediamine-tetra-acetic acid (EDTA) containing tubes and stored at −20°C. DNA was extracted from whole blood using a GoldMag-Mini Whole Blood Genomic DNA Purification Kit (GoldMag Ltd., Xi'an, China) according to the manufacturer's protocol, and the concentration of DNA was measured by NanoDrop 2000C (Thermo Scientific, Waltham, MA). The Sequenom MassARRAY Assay Design 3.0 Software (San Diego, CA) was used to design Multiplexed SNP MassEXTEND assays. PCR primers used for each SNP are listed in Table 7. SNPs were genotyped using Sequenom MassARRAY RS1000 (San Diego, CA) according to the standard protocol recommended by the manufacturer [39]. Polymorphism proliferation was carried out by standard polymerase chain reaction and restriction fragment length polymorphism (PCR—RLFP) as previously described [40]. We applied Sequenom Typer 4.0 Software (San Diego, CA) to perform data management and analyses as described [41].

**Table 7:** PCR primers for SNPs used in this study

SNP	1st_PCRP	2st_PCRP	UEP_SEQ
rs2277698	ACGTTGGATGAGAGT TTATCTACACGGCCC	ACGTTGGATGTCATAC ACACCTGCAATGAG	TTCTTTCCTCC AACGTCCAG
rs2009196	ACGTTGGATGACCTT TCCAGATGTAAGACC	ACGTTGGATGGAGTTA TCCACCTTAAAGGG	AACGTACCCG GCATATTTAG
rs7342880	ACGTTGGATGTGCGG TGCCCGGGAACTAAT	ACGTTGGATGATTCG CCCTGCTTGTCTATG	ccTTCGCCCTGCTT GTCTATGCGATGC
rs11654470	ACGTTGGATGTTCTG CAGGCTCCAGCTTTC	ACGTTGGATGATAAGC AGGACAGGACAGAG	CCCTGCCAGT GCTGGTCCT
rs2003241	ACGTTGGATGGCAAA CTATGGCACAAAGGG	ACGTTGGATGAAATGA AAGGGCGTGGCCAG	GACTCTACAA AAATAGGTGGTG
rs4789936	ACGTTGGATGGCGTC TCACTACCTACAAAG	ACGTTGGATGAACAC ATTACAGGGAGGCAG	aTCTCCAGGGCTG TCTTGAAATCA

### Statistical analysis

Microsoft Excel and SPSS 19.0 statistical packages (SPSS, Chicago, IL) were used to perform statistical analyses. A probability value *p* less than 0.05 was considered statistically significant. Allele frequency of each SNP in the control subjects was analyzed using Chi-squared (χ^2^) test to evaluate departure from Hardy-Weinberg equilibrium (HWE). The χ^2^ test was also used to compare the genotype and allele distributions for SNPs between the control and the knee OA groups [42]. Five genetic models (co-dominant, dominant, recessive, over-dominant, and additive) were applied by PLINK software (http://pngu.mgh.harvard.edu/purcell/plink/). Unconditional logistic regression, adjusted for age and gender, was used to estimate relative risk of knee OA for each of the tested genotype in the form of odds ratio (OR) and 95 % confidence intervals (CIs)[43]. We used Haploview software package (version 4.2) to perform the analysis for linkage disequilibrium (LD), constructed haplotypes and genetic association at significant polymorphism loci.
